# Representation of women in orthopaedic surgery: perception of barriers among undergraduate medical students in Saudi Arabia

**DOI:** 10.1186/s13018-022-03487-6

**Published:** 2023-01-07

**Authors:** Abdulaziz Z. Alomar, Shahd Almonaie, Khalid Nabil Nagshabandi, Deema AlGhufaili, Manar Alomar

**Affiliations:** 1 Head of Arthroscopy and Sports Medicine Division, Orthopaedic Department, College of Medicine, King Saud University, Riyadh, Saudi Arabia; 2 College of Medicine, Alfaisal University, Riyadh, Saudi Arabia; 3 College of Medicine, King Saud University, Riyadh, Saudi Arabia; 4 College of Medicine, King Saud Bin Abdulaziz University for Health Sciences, Riyadh, Saudi Arabia

**Keywords:** Orthopaedic surgery, Medical students, Women surgeons, Female participation

## Abstract

**Background:**

While female participation has improved in several surgical specialties over time globally, no such increase has been observed in orthopaedic surgery over the past decades. The potential barriers to female participation are likely present from the beginning of medical education. Therefore, this study assessed the apparent lag in equal representation among men and women in orthopaedic surgery in the Kingdom of Saudi Arabia.

**Methods:**

This cross-sectional study used a questionnaire survey to investigate medical students’ and interns’ perceptions of women participating in orthopaedic surgery, their subspeciality preferences, and barriers preventing them from pursuing an orthopaedic career. The responses were analysed to understand general perceptions, gender-based differences, impact of clinical experiential learning, and exposure to orthopaedic surgery.

**Results:**

Approximately 565 medical students (49% females, 51% males) participated in the survey. Only 17% of students (11% females, 23% males) considered orthopaedic surgery as their future career option. While 31% of female and 17% of male students disagreed with the concept of female-appropriate orthopaedic subspecialties, most of the remaining male and female students perceived paediatric orthopaedics as a female-appropriate subspecialty. Concerning equal representation of women, gender bias and lack of a strong physique were the most frequently selected barriers by female and male students, respectively. Patient preference for male orthopaedicians, gender discrimination, social and family commitments, and need for physical strength were all perceived as barriers for women in orthopaedics. Overall, clinical experience and orthopaedic exposure did not significantly improve the likelihood of female students in choosing orthopaedic surgery as a career.

**Conclusions:**

The bias against women in orthopaedic careers is prevalent among medical students early in their academic years. Clinical experience and exposure to orthopaedic surgery should be improved to make a significant impact on female participation in orthopaedic careers. Career building efforts in terms of improved career opportunities, career counselling, flexible working hours, social and family related adjustments and implementation of mentorship/research/fellowship programmes for females are needed to reduce gender discrimination and improve female orthopaedic participation. Furthermore, process improvements may yield greater flexibility for women pursuing the challenging field while accommodating other barriers faced by women in orthopaedic surgery.

**Supplementary Information:**

The online version contains supplementary material available at 10.1186/s13018-022-03487-6.

## Introduction

### Background

Surgical specialties have historically witnessed limited female participation [1]; orthopaedic surgery is not untouched by this disparity, exhibiting some of the lowest proportions of female specialty surgeons [2]. Despite the trend of increased interest in the field among women, the proportion of female orthopaedic surgeons is far from representative [2]. The limited involvement of women in orthopaedics could be related to the markedly larger proportion of male surgeons compared to that of females [3, 4]. Women in surgical specialities often face unfavourable work environments exacerbated by personal life pressures that lead to surgeon burnout. Some of the work-related factors include male dominance-related issues accompanied by harassment, performance pressures, and limited support to improve confidence and leadership capacity [5]. Orthopaedics is generally perceived as a specialty requiring physical characteristics that have been traditionally tied to masculinity. Such perceptions may perpetuate outdated ideas of the defining traits of an excellent orthopaedic surgeon, and therefore must be corrected. The true genius in orthopaedics, like many other surgical specialties, lies in the combined application of surgical experience and technological innovations, to deliver effective solutions for improving patient well-being [6, 7]. Decreased participation of women in the field leads to fewer female mentors to guide students and build interest among prospective orthopaedic surgeons early in their medical training. The absence of role models in orthopaedics is, therefore, another barrier that contributes to female dropouts from orthopaedics. There is an observable plateau in the proportion of women pursuing careers in orthopaedics over several years [2]. This decreased participation among women is not an indicator of lesser competence of women as orthopaedic surgeons. Female orthopaedists have been shown to perform equally well as their male colleagues in orthopaedic certification exams [8]. Lack of adequate support from family and peers may exacerbate the poor participation of women, especially considering the vital role of social support in such demanding careers. However, social awareness of gender biases has been changing in pursuit of gender equality. The ratio of female to male medical students is more equalised and female undergraduate medical students perform comparably, if not better than their male counterparts [9]. Moreover, the burden of disease does not discriminate against a single gender and building an inclusive healthcare environment requires inclusiveness in the workplace for all healthcare specialists. Diversity in the workplace has been reported to enhance patient care, improve the accuracy of clinical decision-making, leading to higher patient satisfaction and resulting in improved health outcomes [10].

Improving gender diversity supports effective decision-making, innovation, creativity for organisations, and greater understanding of the patient population [11, 12].

### Rationale

It has been purported that clinical exposure of undergraduate medical students plays an important role in their career decision-making [13]. The length, quality, and nature of the clinical specialty, collectively contribute to student interest in different fields during training [14]. The clinical experiences of undergraduate medical students can, thus, identify the roots of gender bias in the orthopaedic specialty, and guide holistic solutions to build a supportive culture within the specialty for current and future female orthopaedists. Other factors affecting the career decisions of medical students may include experiences shared by colleagues, lifestyle, patient characteristics, family preferences, personal choices, and self-collected information [15]. Considering the many potential factors affecting a woman’s career choice for orthopaedic surgery, it is vital to build a greater understanding of the experiences and perceptions of students early in their medical careers.

Therefore, this study surveyed undergraduate medical students to investigate their perceptions of (1) women in orthopaedic careers, (2) subspeciality preferences among women, (3) barriers to equal representation of women as orthopaedic practitioners and leaders, and (4) influence of experience from general clinical and orthopaedics specialty activities. This study sought to draw deeper insights into the gender bias from the perspective of students who are in the early stages of their medical education. In parallel, the experiential impact of general and specialty clinical practice on such perceptions were reviewed. The study findings can help develop strategic interventions to address gender equality, improve the perception and participation of women in the orthopaedics specialty, and outline a general approach for inclusive professionalism in medical education, postgraduate training, and clinical practice.

## Methods

### Study design and setting

This study followed a cross-sectional design to survey undergraduate medical students during a two-month period from 1 July to 28 August, 2021, after approval from the Institutional Review Board of King Saud University. Informed consent was obtained from all participants prior to participation in the survey.

### Participants

The survey questionnaire was sent through e-mail to all undergraduate medical students (including medical interns) at King Saud University, Riyadh, Kingdom of Saudi Arabia. In the researchers’ institution, students enter the medical phase after 1 year of a preparatory period (pre-medical phase). In the medical phase, the medical curriculum spans across five levels, each with a 1-year duration. Levels 1 and 2 focus on basic sciences, Level 3 is a transition level between preclinical and clinical years; the clinical exposure starts in the third year. Levels 4 and 5 are focused on clinical training. The medical phase is then followed by a 1-year medical internship.

### Exposure to orthopaedic surgery

The students are introduced to orthopaedic surgery in their fourth year during a 4-week outcome-based course exclusively focused on orthopaedics. Several clinical competencies are covered during this period; the students are evaluated through formative, and summative, assessments. The course offers adequate orthopaedic exposure with students scheduled to attend at least three outpatient clinics, three operative room visits, three sessions of working in cast rooms and the emergency department, and optional on-call duties.

### Questionnaire

The survey questionnaire was developed by experts in orthopaedics to understand the perceived gender-based barriers in the field (Additional file 1). The survey questions were constructed through literature review, in relevance to female participation in orthopaedics, and additional inputs from the experts in medical education and orthopaedics. The questionnaire was tested in a small subset of randomly selected undergraduate medical students to ensure that these were clearly interpreted by all the students. Thereafter, the questionnaire’s qualitative content was approved using the Delphi method after mutual consensus was achieved among the review board members.

The questionnaire consisted of 18 questions (Additional file 1). The first four were related to participant sociodemographic background: gender, level of medical education, marital status, and exposure to orthopaedic surgery. The remaining 14 were related to the perception of women participating in orthopaedics. The fifth question was close-ended (‘yes’, ‘no’, or ‘not sure) and enquired about students’ individual preferences toward orthopaedic surgery as a future career. The remaining questions addressed the main objectives of this study related to female representation in orthopaedic surgery. The students’ perception of women in orthopaedic careers was investigated through their opinion on gender diversity in the field of orthopaedic surgery (sixth question) and appropriateness of women in orthopaedic surgery (seventh question) with responses recorded on a five-point Likert scale (wherein, 1 = strongly disagree, 2 = disagree, 3 = unsure, 4 = agree, 5 = strongly agree). The perceived subspeciality preferences among women was assessed using another close-ended question with multiple options regarding need for specific subspecialties for women (eight question). The perception of barriers to equal representation of women as orthopaedic practitioners and leaders was assessed through another close-ended question with multiple options (ninth question) and other questions concerning agreement/disagreement on individual barriers preventing women orthopaedic participation (questions 10–18) used Likert ratings for responses.

### Data collection and statistical analysis

The questionnaire was a mix of seven close-ended questions, and eleven questions with Likert scale-based responses as described above. The survey responses were tabulated in Microsoft Excel (Microsoft Corporation, 2018. Microsoft Excel, https://office.microsoft.com/excel) and individual participant responses were coded to maintain anonymity prior to the analysis. The SPSS software, version 23 (SPSS Inc., Chicago, Illinois, USA) was used for statistical analysis. The sociodemographic characteristics were categorical variables expressed in terms of the number of students and their percentages. Responses to close-ended questions were expressed as frequencies and percentage of individual responses among all students, male–female groups, and groups based on prior exposure to orthopaedic surgery. The percentage distributions of responses were presented in the form of graphical illustrations. The Likert ratings-based responses were expressed as mean among all students and further subgrouping based on male versus female, preclinical versus clinical years, and prior orthopaedic exposed versus unexposed characteristics and their combinations. The orthopaedic course exposed versus unexposed student (only from the clinical years) subgroup was further analysed, considering the confounding influence of clinical exposures from courses, excluding orthopaedics, during their transitional (Level 3) year. These clinical exposures could act as confounders when drawing a comparison between orthopaedic exposed students and all remaining students. The distribution of categorical variables (including frequencies of closed-ended responses) among different groups were compared using the Chi-square test. The overall Likert scale responses for individual questions were analysed using one sample analysis (Wilcoxon signed-rank test based on a hypothesised median of 3) for statistical significance. The Likert scale ratings among different groups and subgroups were compared using Mann–Whitney U-test. All analyses were performed at a significance level of *p* < 0.05.

## Results

A total of 565 students completed the survey. The detailed sociodemographic characteristics are provided in Table 1. While other characteristics were comparable, significantly fewer female students had plans to pursue postgraduate residency training in orthopaedic surgery.

**Table 1:** Sociodemographic characteristics of the study cohort and comparison between male and female students (*n* = 565)

Characteristics	Total number of students, *n* (%)	Male (*n* = 288) number of students (%)	Female (*n* = 277) number of students (%)	*p*-value
*Marital status*				
Single	545 (97)	280 (97)	265 (96)	0.318
Married	20 (3)	8 (3)	12 (4)	
Level*				
Preclinical years	174 (31)	97 (34)	77 (28)	0.130
Clinical years	391 (69)	191 (66)	200 (72)	
*Taken orthopaedic surgery courses*				
Yes	269 (48)	131 (45)	138 (50)	0.303
No	296 (52)	157 (55)	139 (50)	
*Planning to pursue postgraduate residency training in Orthopaedic Surgery*				
Yes	97 (17)	66 (23)	31 (11)	0.001
No	316 (56)	141 (49)	175 (63)	
Do not know	152 (27)	81 (28)	71 (26)	

*Students at individual levels, *n*(%): Level 1: 28 (5), Level 2: 62 (11), Level 3: 84 (15), Level 4: 117 (21), Level 5: 154 (27), medical interns: 120 (21)

### Overall students’ career preferences and perception of gender diversity in orthopaedics

**Fig. 1:**
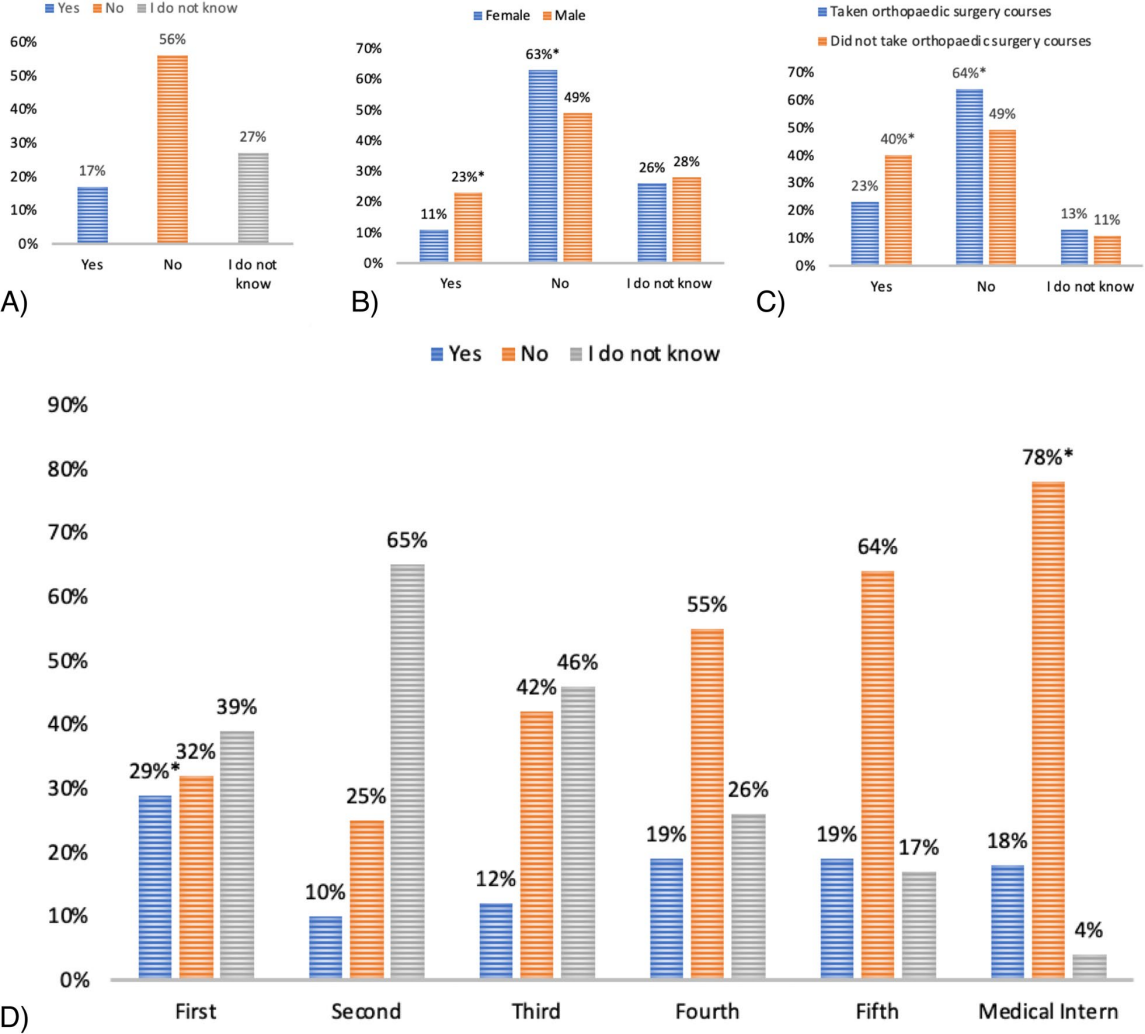
The bar diagrams showing **A** overall interest in an orthopaedic career; **B** gender-based division of orthopaedic career interest, *p-value < 0.05 for males compared to females; **C** orthopaedic surgery exposure-based differences in orthopaedic career interest, *p* < 0.05, *p-value < 0.05 for comparing participants who did and did not take an orthopaedic course; and **D** change in orthopaedic career interest at different academic levels, *p-value < 0.05 for comparisons between academic years

A limited number of students (17%) considered orthopaedics as their future career option while 27% were still unsure of the career option (Fig. 1A). Further subgroup-based analysis revealed a significantly lower interest in orthopaedics among female students while gender differences were insignificant among students unsure of their career option (Fig. 1B). Significantly fewer students with previous exposure to orthopaedics considered orthopaedics as a future career option compared to those without such exposure (Fig. 1C). Orthopaedic surgery exposure did not have any significant impact on students unsure about considering an orthopaedic career. Grouping the responses by level of progression in the medical curriculum showed an increase in uncertainty from year 1–2, followed by a steady decline every year thereafter (Fig. 1D). Only 18% of the medical interns considered orthopaedics as a career option. The Likert scale-based responses suggested that most students agreed with the concept of gender diversity as an important factor contributing to job efficiency in orthopaedics (mean Likert rating: 3.47, *p*-value = 0.001), with significantly higher agreement among male students compared to females (mean Likert ratings: 3.8 vs. 3.16, *p*-value = 0.001). Furthermore, there was an overall disagreement to the concept of females being unfit or inappropriate for orthopaedics or having lesser skills than male orthopaedicians (mean Likert rating: 2.41, *p*-value = 0.001). This disagreement was significantly higher among male students compared to female students (mean Likert ratings: 1.9 vs. 2.89, *p*-value = 0.001).

### Perceived subspeciality preferences for women in orthopaedic careers

**Fig. 2:**
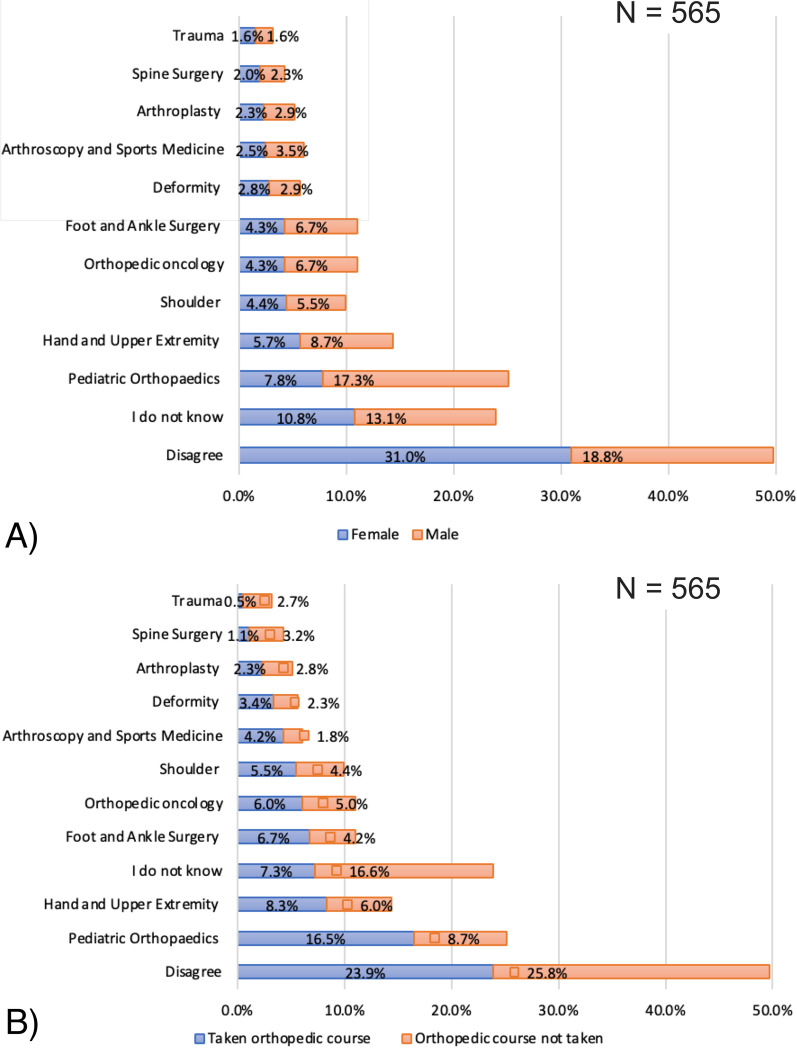
Bar diagrams showing **A** responses of male and female students to the question regarding women-appropriate subspecialties and **B** responses of students who did and did not take an orthopaedic course to the question regarding women-appropriate subspecialties. (*N* = 565 for percentage distribution calculation)

The detailed results are provided in Fig. 2A, B and Table 2. Half of the students disagreed with the question regarding the belief of particular subspecialties in orthopaedic surgery that are fit for of women. Most remaining students suggested paediatric orthopaedics (25%, *p*-value = 0.001) to be the most appropriate and trauma to be the least appropriate subspeciality for women; however, the views on trauma as the least appropriate were statistically insignificant. Subgroup analysis revealed that the proportion of female students disagreeing with women-appropriate subspecialities was significantly higher than their male counterparts (62% vs. 38%, *p*-value = 0.001). In most instances, female students were significantly less likely to suggest subspecialties for women than male students. Paediatric orthopaedics was significantly more frequently suggested by male than female participants as a subspecialty for women (69% vs. 31%, *p*-value = 0.001). Furthermore, participants who took the orthopaedic surgery course, compared with participants who did not, had a significantly higher preference for arthroscopy and sports medicine (71% vs. 29%, *p*-value = 0.006), foot and ankle surgery (61% vs. 39%, *p*-value = 0.022), hand and upper extremity (58% vs. 42%, *p*-value = 0.043), and paediatric orthopaedics (65% vs. 35%, *p*-value = 0.001) as subspecialties that are fit for women.

**Table 2:** Subgroup analysis for percentages of participants who chose subspecialties in orthopaedic surgery that are fit for women

Orthopaedic subspecialties perceived by students that are fit for women (*n* = total times chosen)	Female *N* (%)*	Male *N* (%)*	*P*-value	Yes *N* (%)*	No *N* (%)*	*P*-value
Arthroplasty (*n* = 29)	13 (45)	16 (55)	0.642	13 (45)	16 (55)	0.758
Arthroscopy and Sports Medicine (*n* = 34)	14 (41)	20 (59)	0.345	24 (71)	10 (29)	**0.006**
Deformity (*n* = 32)	16 (50)	16 (50)	0.910	19 (59)	13 (41)	0.170
Disagree (*n* = 281)	175 (62)	106 (38)	**0.001**	135 (48)	146 (52)	0.838
Foot and Ankle Surgery (*n* = 62)	24 (39)	38 (61)	0.085	38 (61)	24 (39)	**0.022**
Hand and Upper Extremity (*n* = 81)	32 (40)	49 (61)	0.064	47 (58)	34 (42)	**0.043**
I do not know (*n* = 135)	61 (45)	74 (55)	0.306	41 (30)	94 (70)	**0.001**
Orthopaedic oncology (*n* = 62)	24 (39)	38 (61)	0.085	34 (55)	28 (45)	0.227
Paediatric Orthopaedics (*n* = 142)	44 (31)	98 (69)	**0.001**	93 (65)	49 (35)	**0.001**
Shoulder (*n* = 56)	25 (45)	31 (56)	0.489	31 (55)	25 (45)	0.221
Spine Surgery (*n* = 24)	11 (47)	13 (53)	0.749	6 (25)	18 (75)	**0.023**
Trauma (*n* = 18)	9 (49)	9 (51)	0.933	3 (17)	15 (83)	**0.008**

*p* < 0.05 are shown in bold

*N: number of participants who chose the corresponding option as a subspecialty in orthopaedic surgery that are fit for women

Percentage represents number of participants from the total times the corresponding subspeciality was chosen

Yes: Taken orthopaedic surgery course

No: No orthopaedic surgery course taken

### Perceptions of barriers to representation of women in orthopaedic careers

**Fig. 3:**
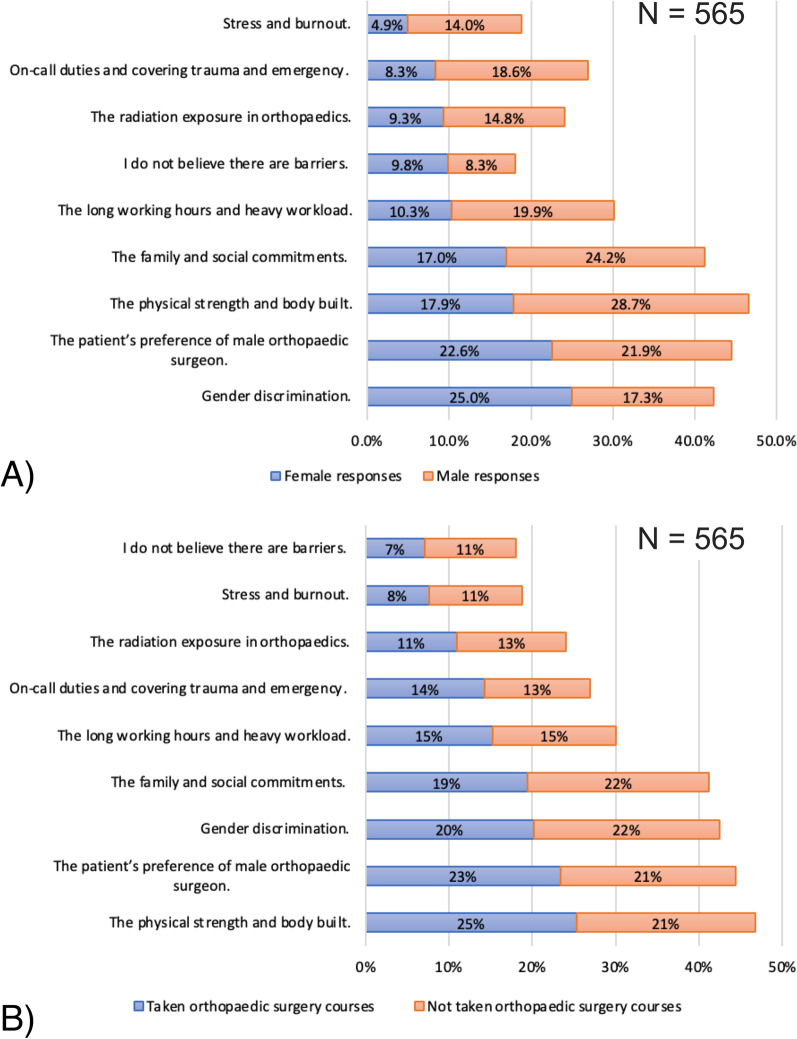
Bar diagrams showing **A** male versus female distribution of students’ perception regarding barriers for women seeking orthopaedic careers and **B** orthopaedic course exposure-based subgrouping of all students’ perceptions regarding barriers for women in orthopaedic careers. (*N* = 565 for percentage distribution calculation)

The detailed results are provided in Fig. 3A, B and Table 3. The overall responses to the factors preventing females from seeking an orthopaedic career revealed that 81.9% of the students believed there were barriers to female representation in orthopaedics. Moreover, gender-based subgrouping of responses revealed that 83.6% of the male and 80.1% of the female students perceived such barriers. The need for job appropriate physical characteristics (46.5%), patient preference for male orthopaedicians (44.4%), elements of gender discrimination (42.4%), and social/family commitment (41.2%) were the top perceived barriers to female participation in orthopaedics among both male and female subgroups. Female students felt discriminated against owing to these top barriers (59% vs. 41%, *p*-value = 0.001), while males felt physical strength/body build to be the top barrier (62% vs. 38%, *p*-value = 0.001). These perceptions persisted among students after taking the orthopaedic course. More students who took orthopaedic courses perceived gender roles in on-call duties, patient preferences toward male orthopaedicians (53% vs. 47%, *p*-value = 0.034), and the need for better physical build (54% vs. 46%, *p*-value = 0.003) to be the barriers for women participation in orthopaedics.

**Table 3:** Subgroup analysis for percentages of participants who chose the top barriers for females in orthopaedic surgery

Barriers for women in orthopaedic surgery (*n* = total times chosen)	Female *N* (%)*	Male *N* (%)*	*P*-value	Yes *N* (%)*	No *N* (%)*	*P*-value
Gender discrimination (*n* = 240)	142 (59)	98 (41)	**0.001**	114 (48)	126 (52)	0.964
I do not believe there are barriers (*n* = 102)	55 (54)	47 (46)	0.275	40 (39)	62 (61)	0.061
On-call duties and covering trauma and emergency (*n* = 152)	47 (31)	105 (69)	**0.001**	81 (53)	71 (47)	0.101
Stress and burnout (*n* = 106)	28 (26)	78 (74)	**0.001**	43 (41)	63 (59)	0.107
The family and social commitments (*n* = 233)	96 (41)	137 (59)	**0.002**	110 (47)	123 (53)	0.873
The long working hours and heavy workload (*n* = 170)	58 (34)	112 (66)	**0.001**	86 (51)	84 (49)	0.353
The patient’s preference of male orthopaedic surgeon (*n* = 251)	127 (51)	124 (49)	0.504	132 (53)	119 (47)	**0.034**
The physical strength and body build (*n* = 264)	101 (38)	163 (62)	**0.001**	143 (54)	121 (46)	**0.003**
The radiation exposure in orthopaedics (*n* = 136)	53 (39)	83 (61)	**0.005**	62 (46)	74 (54)	0.588

*p* < 0.05 are shown in bold

*N: number of participants who chose the corresponding option as a barrier for women practicing in orthopaedic surgery

Percentage represents number of participants from the total times the corresponding barrier was chosen (i.e. ‘*n*’ specified in first column)

Yes: Taken orthopaedic surgery course

No: No orthopaedic surgery course taken

Subgroup analysis of male and female responses revealed that females more frequently identified stress and burnout, inferior orthopaedic surgical abilities, inappropriateness of the field for women, and physical and social concerns as barriers preventing a greater representation of women in orthopaedics. However, males had higher agreement to gender discrimination in orthopaedics. The details are presented in Table 4.

**Table 4:** Perception of medical students regarding barriers to women in orthopaedics and the associated impact of gender

Question	Overall Likert ratings		Gender-based differences		
	Mean Likert ratings	Wilcoxon Signed-rank test *p*-value	Female students mean Likert ratings	Male students mean Likert ratings	Mann–Whitney U-test *p*-value
I believe that patients have more confidence in male orthopaedic surgeons than females	3.58	**0.001**	3.68	3.48	0.131
I believe that the radiation exposure risk in orthopaedics can create a barrier for women practicing in orthopaedic surgery	2.79	**0.001**	2.99	2.58	**0.001**
I believe that the on-call duties and covering trauma and emergency in orthopaedics can create a barrier for women practicing in orthopaedic surgery	2.95	0.345	3.25	2.65	**0.001**
I believe that gender diversity in the field of orthopaedic surgery is an important factor contributing to the job efficiency and the community of orthopaedic surgery	3.47	**0.001**	3.16	3.8	**0.001**
I believe that in orthopaedic surgery the gender discrimination exists against women and can be a barrier for them during the practice	3.59	**0.001**	3.24	3.94	**0.001**
I believe that the orthopaedic surgical abilities of male surgeons are superior to the abilities of female orthopaedic surgeons	2.48	**0.001**	3.05	1.89	**0.001**
I believe that orthopaedic surgery does not fit and is not appropriate for females	2.41	**0.001**	2.89	1.91	**0.001**
I believe that ‘family and social commitments’ create a barrier for women practicing in orthopaedic surgery	3.21	**0.001**	3.45	2.96	**0.001**
I believe that the ‘physical strength and body build’ is important for practicing orthopaedic surgery and it can create a barrier for women practicing in orthopaedic surgery	3.45	**0.001**	3.7	3.2	**0.001**
I believe that the ‘long working hours and the heavy workload’ can create a barrier for women practicing in orthopaedic surgery	2.99	0.793	3.13	2.71	0.192
I believe that the ‘stress and burnout’ in orthopaedics can create barriers for women in orthopaedic surgery	2.78	**0.001**	3.06	2.49	**0.001**

*p* < 0.05 are shown in bold

### Influence of clinical and orthopaedic exposure on student perceptions

#### Impact of the progress in the medical curriculum on student perceptions

The level progression did not significantly affect the students’ perceptions of barriers for women in orthopaedics, except for stress and burnout in orthopaedics. The female students in their clinical years had a significantly higher agreement on patient preference for male orthopaedic surgeons and gender discrimination in orthopaedics. Such an effect was not significantly prevalent among male students. The detailed results are provided in Table 5.

**Table 5:** Impact of levels and clinical years on students’ perceptions

Question	Kruskal–Wallis test *p*-value for trend	Overall Likert ratings			Female students Likert ratings			Male students Likert ratings		
		Preclinical year (*N* = 174) (mean)	Clinical year (*N* = 391) (mean)	Mann–Whitney U-test *p*-value	Preclinical (*N* = 77) (mean)	Clinical (*N* = 200) (mean)	Mann–Whitney U-test *p*-value	Preclinical (*N* = 97) (mean)	Clinical (*N* = 191) (mean)	Mann–Whitney U-test *p*-value
I believe that patients have more confidence in male orthopaedic surgeons than females	0.051	3.39	3.67	**0.008**	3.19	3.58	**0.013**	3.55	3.75	0.149
I believe that the radiation exposure risk in orthopaedics can create a barrier for women practicing in orthopaedic surgery	0.056	2.79	2.79	0.993	2.49	2.61	0.41	3.03	2.97	0.754
I believe that the on-call duties and covering trauma and emergency in orthopaedics can create a barrier for women practicing in orthopaedic surgery	0.374	2.86	2.99	0.226	2.56	2.68	0.388	3.09	3.32	0.159
I believe that gender diversity in the field of orthopaedic surgery is an important factor contributing to the job efficiency and the community of orthopaedic surgery	0.209	3.4	3.5	0.309	3.58	3.89	0.072	3.26	3.1	0.342
I believe that in orthopaedic surgery the gender discrimination exists against women and can be a barrier for them during the practice	0.055	3.53	3.6	0.278	3.77	4.01	**0.046**	3.55	3.18	0.339
I believe that the orthopaedic surgical abilities of male surgeons are superior to the abilities of female orthopaedic surgeons	0.978	2.48	2.48	0.945	1.87	1.89	0.719	2.97	3.09	0.521
I believe that orthopaedic surgery does not fit and is not appropriate for females	0.688	2.29	2.46	0.151	1.7	1.99	0.107	2.76	2.95	0.277
I believe that ‘family and social commitments’ create a barrier for women practicing in orthopaedic surgery	0.29	3.25	3.19	0.573	2.86	3.01	0.387	3.56	3.39	0.205
I believe that the ‘physical strength and body build’ is important for practicing orthopaedic surgery and it can create a barrier for women practicing in orthopaedic surgery	0.228	3.32	3.51	0.108	3.03	3.27	0.177	3.56	3.77	0.159
I believe that the ‘long working hours and the heavy workload’ can create a barrier for women practicing in orthopaedic surgery	0.131	2.93	3.02	0.405	2.7	2.75	0.78	3.1	3.3	0.214
I believe that the ‘stress and burnout’ in orthopaedics can create barriers for women in orthopaedic surgery	**0.031**	2.79	2.77	0.997	2.43	2.51	0.546	3.07	3.05	0.933

*p* < 0.05 are shown in bold

#### Overall impact of orthopaedic surgery exposure

Compared to students without exposure, those exposed to orthopaedic surgery were more likely to perceive that women were not the best fit for orthopaedic surgery. Concerns that physical strength, body physique, and workload were barriers to women participating in orthopaedics were also strengthened after orthopaedic course exposure. However, the perceptions of barriers among female students exposed to the orthopaedic course did not significantly differ from those who were not exposed. Male students exposed to orthopaedic surgery, however, had a significantly higher agreement to the inappropriateness of orthopaedics for women as well as the barriers of physical strength and build, workload, long working hours, and on-call duties. The details are presented in Table 6.

**Table 6:** Overall impact of the orthopaedic course on medical students’ perceptions regarding barriers for women in orthopaedic careers and the associated impact of gender

Question	Overall Likert ratings Took orthopaedic course (Yes/No)			Female students Likert ratings Took orthopaedic course (Yes/No)			Male students Likert ratings Took orthopaedic course (Yes/No)		
	Yes (*N* = 269) (mean)	No (*N* = 296) (mean)	Mann–Whitney U-test *p*-value	Yes (*N* = 138) (mean)	No (*N* = 139) (mean)	Mann–Whitney U-test *p*-value	Yes (*N* = 131) (mean)	No (*N* = 157) (mean)	Mann–Whitney U-test *p*-value
I believe that patients have more confidence in male orthopaedic surgeons than females	3.65	3.52	0.152	3.53	3.42	0.496	3.79	3.6	0.141
I believe that the radiation exposure risk in orthopaedics can create a barrier for women practicing in orthopaedic surgery	2.76	2.81	0.635	2.59	2.57	0.672	2.94	3.03	0.632
I believe that the on-call duties and covering trauma and emergency in orthopaedics can create a barrier for women practicing in orthopaedic surgery	3.06	2.85	0.054	2.75	2.55	0.172	3.39	3.13	0.078
I believe that gender diversity in the field of orthopaedic surgery is an important factor contributing to the job efficiency and the community of orthopaedic surgery	3.49	3.46	0.679	3.87	3.73	0.297	3.09	3.21	0.41
I believe that in orthopaedic surgery the gender discrimination exists against women and can be a barrier for them during the practice	3.62	3.54	0.282	3.99	3.89	0.223	3.24	3.24	0.937
I believe that the orthopaedic surgical abilities of male surgeons are superior to the abilities of female orthopaedic surgeons	2.54	2.42	0.253	1.93	1.85	0.568	3.19	2.93	0.105
I believe that orthopaedic surgery does not fit and is not appropriate for females	2.53	2.29	**0.043**	1.97	1.84	0.669	3.11	2.69	**0.003**
I believe that ‘family and social commitments’ create a barrier for women practicing in orthopaedic surgery	3.23	3.2	0.771	3.01	2.92	0.568	3.46	3.44	0.939
I believe that the ‘physical strength and body build’ is important for practicing orthopaedic surgery and it can create a barrier for women practicing in orthopaedic surgery	3.55	3.37	**0.043**	3.2	3.19	0.858	3.91	3.53	**0.003**
I believe that the ‘long working hours and the heavy workload’ can create a barrier for women practicing in orthopaedic surgery	3.12	2.86	**0.01**	2.86	2.61	0.103	3.4	3.09	**0.024**
I believe that the ‘stress and burnout’ in orthopaedics can create barriers for women in orthopaedic surgery	2.84	2.72	0.199	2.58	2.4	0.201	3.11	3.01	0.355

*p* < 0.05 are shown in bold

#### Impact of taking an orthopaedic course among clinical year students

Subgroup analysis was performed between clinical year students who were exposed to orthopaedic and clinical year students who had not begun their orthopaedic course to control the confounding influence of clinical exposures other than orthopaedics. After orthopaedic course exposure, participants showed significantly higher agreement to the perception that stress and burnout (*p* = 0.018), on-call duties and coverage of trauma and emergency (*p* = 0.015), and long working hours and heavy workload (*p* = 0.001) were barriers for women in considering an orthopaedic career (Table 7).

**Table 7:** Change in clinical year students’ perceptions after orthopaedic course exposure

Questions	Likert ratings Clinical year students exposed to orthopaedic course (Yes/No)		Mann–Whitney U-test *p*-value
	Yes (*N* = 265) (mean)	No (*N* = 210) (mean)	
I believe that patients have more confidence in male orthopaedic surgeons than females	3.69	3.53	0.123
I believe that the radiation exposure risk in orthopaedics can create a barrier for women practicing in orthopaedic surgery	2.75	2.72	0.823
I believe that the on-call duties and covering trauma and emergency in orthopaedics can create a barrier for women practicing in orthopaedic surgery	3.06	2.79	**0.015**
I believe that gender diversity in the field of orthopaedic surgery is an important factor contributing to the job efficiency and the community of orthopaedic surgery	3.49	3.54	0.776
I believe that in orthopaedic surgery the gender discrimination exists against women and can be a barrier for them during the practice	3.64	3.56	0.362
I believe that the orthopaedic surgical abilities of male surgeons are superior to the abilities of female orthopaedic surgeons	2.56	2.36	0.095
I believe that orthopaedic surgery does not fit and is not appropriate for females	2.54	2.31	0.083
I believe that ‘family and social commitments’ create a barrier for women practicing in orthopaedic surgery	3.24	3.19	0.574
I believe that the ‘physical strength and body build’ is important for practicing orthopaedic surgery and it can create a barrier for women practicing in orthopaedic surgery	3.57	3.41	0.062
I believe that the ‘long working hours and the heavy workload’ can create a barrier for women practicing in orthopaedic surgery	3.12	2.76	**0.001**
I believe that the ‘stress and burnout’ in orthopaedics can create barriers for women in orthopaedic surgery	2.84	2.6	**0.018**

*p* < 0.05 are shown in bold

## Discussion

### Background and rationale

The key findings of this study suggest that the perceptions associated with a limited representation of women in orthopaedics exist from the beginning of undergraduate medical education. The desire among female medical students to pursue orthopaedics as a future career was statistically suppressed by several physical, mental, and social barriers resulting in reduced interest as they progressed in their medical education. While most students felt that gender diversity is required for the efficient functioning of the department—irrespective of their gender, seniority, and prior exposure to orthopaedics—the clinical environment and the orthopaedic courses have not adequately addressed this concern. This observation is supported by the study findings, which demonstrate that female students have a statistically higher agreement regarding the presence of gender discrimination and lack of patient support during their clinical years and that these perceptions do not change even after exposure to orthopaedic surgery. Therefore, due to this lack of adequate female representation in orthopaedics, the current study, with its roots at the beginning of undergraduate medical education, presents deeper insight into the problem and the potential solutions for it. This study suggests how medical students in the Kingdom of Saudi Arabia perceive different physical and social barriers that prevent female participation in orthopaedics.

### Limitations

This study has some limitations. First, students’ perceptions were recorded and were found relevant to the female representation in orthopaedics as a career for only a fixed number of issues. Therefore, other relevant aspects might have been missed in this study’s analysis. Second, the current study surveys the perceptions of medical students a medical school in the Kingdom of Saudi Arabia, which could have been influenced by the social practices of the region or population. Such differences are evident from past studies on regional gender variations in surgical specialties [16, 17]. Thus, female orthopaedic career opportunities may be skewed by the different issues of different regions. Third, the results can be affected by the institutional practices relevant to medical education, clinical training and orthopaedic exposure, and mentorship, which may vary among different institutions. Fourth, this study only highlights the issues faced by the female students that prevent them from opting for orthopaedic careers; it does not investigate the institutional policies and practices that affect such perceptions. The origin of such perceptions is debatable and can be influenced by the local population, institution, type of orthopaedic exposure, and mentorship programmes, which was beyond the scope of this study. This would require separate extensive research to objectively measure the source and factors leading to students’ perceptions of women in orthopaedics. Lastly, this study could not conclude the failure of clinical and orthopaedic exposure in changing students’ perceptions; this is because these perceptions, before and after such exposures, were not compared for the same student group. A prospective study is warranted to investigate this further.

### Women in orthopaedic careers

The current evidence suggests that female representation is almost equal to that of males in most undergraduate medical schools [9]. However, women constitute less than a percent of the orthopaedic residents around the world, despite a marked rise in female representation in non-surgical specialties [2]. While this could be attributed to multiple factors, the lack of same-gender role models in orthopaedic specialties is a major factor that results in female difficulty in channelising their interest into a real career option [9, 18]. Thus, better representation of the female faculty in the orthopaedics department is crucial to introduce interest for orthopaedic careers among female medical students, which would encourage female students to connect with them more comfortably. There is evidence that surgical departments with higher female faculties have better female trainee representation [19].

### Subspeciality preferences among women

The female students did not have any inclination towards particular subspecialties, as most females (a higher proportion than males) disagreed with the concept of women-appropriate subspecialties. Among the remaining students, paediatric orthopaedics and upper limb and hand were preferred by most. Therefore, these findings are in line with most observations in the literature on female orthopaedic trainees and practitioners’ perceptions. Rohde et al. [12] and Bratescu et al. [20], in separate surveys of female orthopaedic members of the Ruth Jackson Orthopaedic Society, found that hand (24% in both surveys) and paediatric orthopaedics (19%, 22.6%, respectively) were the most preferred subspecialties. Similarly, Hariri et al. [21] reported that a significantly higher proportion of women pursue paediatric and hand fellowships as compared to men (24% vs. 6% and 20% vs. 13%, respectively) and that more men were planning to pursue a sports fellowship compared to women (31% vs. 11%). Cannada [8] observed that the highest proportion of women trainees (25%) was opting for paediatric orthopaedic fellowships, followed by foot and ankle (14%), while spine had the lowest proportion (3%). Besides paediatric orthopaedics, sports medicine is among the top women-preferred specialties in some studies [8, 12, 20]. Although only 6% of the students suggested arthroscopy and sports as women-appropriate specialties, a significant improvement was observed after exposure to the orthopaedic course. Trauma and spine were the least preferred specialties among the medical students in this study. The exposure to orthopaedic surgery further strengthened these perceptions. Hence, these findings are also supported by most studies on female orthopaedic trainees’ and practitioners’ interests [8, 12, 20]. These preferences are likely to correlate with barriers rather than competence.

### Barriers to equal representation of women as orthopaedic practitioners and leaders

Strong mentorship in medical school, interest in manual tasks, professional satisfaction, intellectual stimulation, work/life balance, and perception regarding inadequate physical strength are some factors affecting the students’ decision-making process [12, 20]. Brook et al. [22] observed that mentoring opportunities are inadequate in medical schools. However, Jurenovich et al. [23] observed that 79% of the female trainees did not find female mentorship, family, pregnancy, significant other, or physical attributes as factors that influence their fellowship preferences; a year’s duration of fellowship could be the possible reason and the overall influence of mentorship on a long-term career might have been different. Currently, there is no evidence to support gender influence on surgical skills among the specialty trainees or residents. Gender discrimination is a worldwide concern and one of the major barriers to female participation in orthopaedics [24]. More than two-fifths of students (most of them being female) witnessed gender discrimination in orthopaedics; even the male students expressed a strong agreement with this. Discrimination can take the form of sexual harassment as well, which includes inappropriate physical contact as well as derogatory remarks and behaviour [25, 26]. Such incidents may be detrimental to female representation in orthopaedics and are often underreported. Educating the students and trainees on gender bias and discrimination, discriminatory behaviours, and intimidating verbal and non-verbal actions, and improving female recruitment and retention to address and minimise such incidents would be a significant step toward an improved and healthy workplace environment.

Family and social commitments can act as barriers to female participation in orthopaedics. This perception was agreed upon by almost two-fifths of the students, with comparable responses between males and females. Women often have domestic responsibilities, such as taking care of the home and children, which may make it difficult for them to maintain a work-life balance in demanding surgical fields—especially those involving long and odd working hours, on-call duties, physical workload, stress, and burnout. In this study findings, the perception regarding working hours, on-call duties, stress, and burnout as barriers was almost neutral, although with higher agreement among females.

The perception regarding working hours, on-call duties, stress, and burnout as barriers to female orthopaedic participation was even stronger in males who were exposed to orthopaedic courses. Additionally, the perceptions regarding on-call duties, trauma and emergency coverage in orthopaedics, long working hours and the heavy workload, and stress and burnout had a significantly stronger concordance among clinical year students who underwent the orthopaedic course compared to the remaining clinical year students. These findings suggest that current orthopaedic surgery practices pose a barrier to female participation, in addition to the social and domestic commitments. Previous studies have reported similar findings. Hariri et al. [21], in a survey of orthopaedic residents, found that significantly more women planned to change their work status to ‘part-time’ or reduce their work hours compared to men. Amoli et al. [27] found that the women orthopaedicians’ weekly workload and surgical case volume was lower than that of men, and 26% of the men reported performing more than seven surgeries per week compared to 10% of the women. Madhuri et al. [28], in a survey of women orthopaedicians, found that maintaining work-life balance was considered challenging for 40% of the women, and the inability to achieve full working potential was reported by 60% of the women. In a study conducted by Klein et al. [29], it was reported that 75% of the orthopaedic surgeons did not have adequate time to attend to their personal lives. The career of a female orthopaedist can also be affected by their productivity as faculty members, marital life, and parenthood. As these factors are difficult to modify, an individualised approach would be required to promote job satisfaction among female orthopaedic aspirants. The orthopaedic subspecialties can provide a tailored experience for women. This was evident from the females considering paediatric orthopaedics as the most desirable option. Childbearing can also impact the physical capabilities of women, for which appropriate work-related accommodation should be provided, and medical students should be subsequently informed so that it does not limit them from joining orthopaedics. In this study, very few students were married and none of them had children. Thus, the impact of these factors on women participation in orthopaedics could not be analysed.

Nearly one-fourth of the students had concerns regarding radiation exposure as a barrier to female participation in orthopaedics. The overall agreement was almost neutral, with but significantly higher perception among females. However, it must be emphasised that the radiation exposure risks remain same for men and women, except during pregnancy. The foetus is most vulnerable in the first trimester with the organ malformations only occurring with a highly concentrated exposure to radiation of more than 100 mGy. However, with available radiation protection equipment, the risk to pregnant women is insignificant and thus training and practice opportunities can be provided to pregnant women [30]. Finally, there is the issue of the popular perception of orthopaedicians as ‘strong as an ox and just as smart’, which could be a myth and should be disregarded in current scenarios [7]. No human has the same physical strength, and this would apply to orthopaedics as well. Several authors advocated for correct use of body mechanics rather than the physical strength as the requirement in orthopaedics, and nobody ruled out orthopaedic surgery based on solely their body build or physical strength [31, 32]. Moreover, since it is rare to perform a major physical task without the assistance of a team, the perception regarding physical strength as a barrier to selecting orthopaedics as a career choice is obsolete. Based on findings in the available literature, mentorship programmes for female medical students are needed to encourage them toward orthopaedic careers [33]. Additionally, there is a need for attractive job opportunities and their awareness among women orthopaedicians at major faculty positions, while accommodating their family and social responsibilities [34]. Such women can act as potential role models for female medical students. Further, strict surveillance of operating rooms and clinics’ activities to monitor incidents of gender discrimination and harassment must be implemented so that orthopaedic practices are in line with standard protocols. Medical students should not be made to think that orthopaedics is labour-intensive job. Anonymous reporting systems should be developed to monitor discriminatory activities and associated codes of conduct [34]. Efforts should be made to encourage active participation of female medical students in orthopaedic departmental activities. Special career counselling measures may also be needed to ensure that female students are aware of all options in orthopaedic surgery and choose their career wisely.

### Influence of exposure to clinical and orthopaedic practice on student perceptions

Although early clinical exposure has been introduced in medical education worldwide, there is still a need for better designing of career development activities right from the beginning—that is, from the preclinical years, when students are inclined toward a particular career that is based on what they have heard from others rather than on their own experiences. This state of uncertainty is evident from the present findings, which revealed that a higher number of second year students were unsure of their careers compared to the first year students. A higher number of male students agreed with the inappropriateness of orthopaedics for women after their exposure to orthopaedic courses. Notably, the clinical years and orthopaedic surgery exposures have failed to change this neutral perception to a positive one. Since the preclinical experience encompasses a long period, the students might not be in a state of mind to change their career options by the time they reach the clinical phase, even if they get better clinical opportunities. The inclusion of research and academic opportunities during the early years can help students get involved with the departments, thus helping in their future career planning [35]. Moreover, there is a need for special career counselling cells in medical schools that can provide expert advice and respond to career-related queries of the medical students. The problem of limited female representation in orthopaedics has attracted medical experts’ attention. Consequently, some associations are currently helping in providing mentorship, research, and fellowship opportunities to female students [12]. Strengthening such initiatives at the medical school level can potentially help recruit more female orthopaedic surgeons. Furthermore, there is a probability that the issues discussed in this study have contributed to reduced interest among females toward orthopaedics as a career (11%), despite most of them having taken orthopaedic courses. On the contrary, more than half of the male students, who attended the orthopaedic course, showed interest in orthopaedics as a career option. This study demonstrated that exposure to the current orthopaedic programme did not significantly improve the consideration of orthopaedics as a career among female participants, thereby obviating the need for more effective clinical programmes that result in more positive perceptions and encourage greater female participation in orthopaedics. Although the aforementioned barriers to women’s orthopaedic career persisted despite orthopaedic course exposure, other stronger negative perceptions related to stress and burnout, on-call duties, covering trauma and emergency, and long working hours and heavy workload—which discourage women from joining orthopaedics—is a matter of concern. In addition, a limited representation of female orthopaedic faculty during student training could have adversely impacted students’ perceptions of female participation in orthopaedics; for instance, currently, there is only one female orthopaedic surgeon at King Saud University. The students might never have witnessed women orthopaedicians, who have social and family responsibilities, working in the department or been informed about the policies and accommodations for women in orthopaedics. This could have strengthened their perception that orthopaedic surgery is not an ideal field for women. Since this issue is linked with social obligations, students’ clinical years and orthopaedic exposure could not change their perception of social and family commitments as barriers for women in orthopaedics.

Overall, the students’ perception of patients’ preference for male orthopaedic surgeons has not been supported by the current literature on patient preferences. The published evidence suggests that most patients do not have any gender preferences for orthopaedic surgeons [31, 36]. However, some patients do prefer same-gender orthopaedicians. For example, Dineen et al. [31] observed that some patients preferred female orthopaedicians for paediatric consultations and hand surgery, and male orthopaedicians for arthroplasty and spine surgeries; however, these findings were statistically insignificant. This study’s observations suggest that students’ perception of patients’ male preference sets in prior to their clinical exposure, after which it only becomes stronger. However, the observations regarding gender preferences in some subspecialties were statistically insignificant. Contradicting gender preferences, Errani et al. [33] observed in their literature review that women physicians tend to have more empathy toward their patients, spend more time with them, and are more sensitive than male physicians. Unlike the findings of aforementioned studies, Fink et al. [37] observed that both male and female patients often preferred to see a same-gender primary care physician; this preference is more pronounced among males. While these findings are supportive toward female orthopaedicians, patient profile, gender, literacy levels, and social environments may also have regional variations that affect such attitudes. Therefore, in this study, the students most likely held this perception prior to clinical exposure owing to current trends in society, and this perception became stronger after clinical exposure. Moreover, a nonuniform proportion of male versus female patients in orthopaedic clinics, during different clinical exposures, could also have contributed to the varying experiences. Patients’ education level and social awareness could help them in developing positive gender perceptions. Surgical abilities reflect the way residents have been trained irrespective of their gender or background [6]. This study’s findings suggest that most female students held the perception that male orthopaedicians have better surgical abilities, which could reflect the preexisting male dominance in orthopaedics. However, with clinical exposure, the response toward this perception became mostly neutral. This, again, highlights the need for early inclusion of career development opportunities, which can clear these misconceptions through tangible evidence. Of the most discussed barriers which persisted after the orthopaedic course, a deficiency of established female orthopaedic faculty and mentors could potentially influence female students’ perception toward orthopaedic surgery careers and they may feel that they are not appropriate for it [18]. This study’s findings suggest that there was constant agreement regarding the need for physical build and strength in orthopaedics even after clinical and orthopaedic course exposure. This might be because of preexisting misconceptions and lack of knowledge about the subspecialties that suit individual demands and the development of tools that make tasks easier. The students might not be able to obtain deep insights into orthopaedic specialty in a short period, which may require a longer clinical exposure in orthopaedics or special career counselling measures. In addition, this may be due to the ongoing orthopaedic surgical practices and manoeuvres, or the way in which they are presented to medical students. For example, the male dominant department might be performing the tasks as per their interest, potentially involving displays of strength. Further, other tools or methods which are available to facilitate tasks might not have been used. Fram et al. [38], in a survey among female orthopaedicians, observed that they face difficulty in using several common orthopaedic surgical instruments unlike their male counterparts. Such perceptions can develop among medical students as they observe surgical procedures. The orthopaedic course did not significantly improve students’ neutral perception regarding discrimination against women in orthopaedics and that regarding gender discrimination persisted even after orthopaedic surgery exposure and clinical years, which suggests a lack of meaningful impact; however, female students perceived these issues more strongly after their clinical years. To some extent, it may relate to male dominance and lack of female-appropriate behaviour to which the male members might not be accustomed. Moreover, habitual communication from male colleagues may be perceived as discriminatory behaviour. Several surveys have reported that the elements of discrimination, bullying, and sexual harassment negatively impact women’s interest in orthopaedics [3]. A negative attitude toward females as orthopaedic colleagues can also impact their orthopaedic career interest. Bucknall et al. [3], in a survey of medical students, noted that most students were told by experienced professionals that female surgeons and family life should never coincide, and few were told that only men should undertake surgery as women did not possess skills and strength to competently operate. In a recent study by Rahman et al. [16], the authors compared the perceptions regarding diversity and inclusion among different demographic groups of medical students, which included genders, races or ethnicities, and sexual orientations. The authors found a general improvement in all demographic groups concerning the students’ perception regarding diversity and inclusiveness after orthopaedic exposure. Similar to the findings of this study, prior to orthopaedic exposure, most female students believed orthopaedics to be less diverse, less inclusive, gender discriminatory, labour-intensive, and an unlikely career option for themselves. While this study projected that orthopaedic exposure helps in mitigating several negative perceptions among diverse students, some notable issues persisted even after orthopaedic exposure. For instance, when compared to their male counterparts, female students were still highly unlikely to pursue an orthopaedic job, both before and after orthopaedic exposure, and expressed strong agreement toward the perception regarding the job being labour-intensive. However, the in-depth insights on individual factors contributing to such persistence were not included in this study. The study findings highlight the aspects which potentially result in negative perceptions among female students regarding an orthopaedic career.

## Conclusion

The perception of women being unfit for orthopaedic surgery careers prevails among medical students from their early academic years. Medical education, clinical exposure, and exposure to orthopaedic surgery must be improved to encourage women to pursue orthopaedic surgery as a valid career option. Supportive measures related to orthopaedic careers for women should be implemented by reducing gender discrimination, improving career counselling, providing mentorship/research/fellowship programmes for females in orthopaedics, and making provisions to address the family and social commitments of women.

## Supplementary Information

**Additional file 1.** Questionnaire to investigate undergraduate medical students’ perception of women participating in orthopaedic careers.

## Data Availability

All data related to this study are available and ready upon request from the author.

## References

[1] Blakemore LC, Hall JM, Biermann JS (2003). Women in surgical residency training programs. J Bone Joint Surg Am.

[2] Brotherton SE, Etzel SI (2018). Graduate medical education, 2017–2018. JAMA.

[3] Bucknall V, Pynsent PB (2009). Sex and the orthopaedic surgeon: a survey of patient, medical student, and male orthopaedic surgeon attitudes towards female orthopaedic surgeons. Surgeon.

[4] Munger AM, Heckmann N, McKnight B, Dusch MN, Hatch GF, Omid R (2019). Revisiting the gender gap in orthopaedic surgery: Investigating the relationship between orthopaedic surgery female faculty and female residency applicants. J Am Acad Orthop Surg.

[5] Lim WH, Wong C, Jain SR, Ng CH, Tai CH, Devi MK, Samarasekera DD, Iyer SG, Chong CS (2021). The unspoken reality of gender bias in surgery: a qualitative systematic review. PLoS ONE.

[6] Ali A, Subhi Y, Ringsted C, Konge L (2015). Gender differences in the acquisition of surgical skills: a systematic review. Surg Endosc.

[7] Subramanian P, Kantharuban S, Subramanian V, Willis-Owen SA, Willis-Owen CA (2011). Orthopaedic surgeons: as strong as an ox and almost twice as clever? Multicentre prospective comparative study. BMJ.

[8] Cannada LK (2016). Women in Orthopaedic Fellowships: What is their match rate, and what specialties do they choose?. Clin Orthop Relat Res.

[9] Hill JF, Yule A, Zurakowski D, Day CS (2013). Residents’ perceptions of sex diversity in orthopaedic surgery. J Bone Joint Surg Am.

[10] Gomez LE, Bernet P (2019). Diversity improves performance and outcomes. J Natl Med Assoc.

[11] Nielsen MW, Alegria S, Börjeson L, Etzkowitz H, Falk-Krzesinski HJ, Joshi A, Leahey E, Smith-Doerr L, Woolley AW, Schiebinger L (2017). Opinion: gender diversity leads to better science. Proc Natl Acad Sci USA.

[12] Rohde RS, Wolf JM, Adams JE (2016). Where are the women in orthopaedic surgery?. Clin Orthop Relat Res.

[13] Coffeng LE, Visscher AJ, Ten Cate OT (2009). The influence of early clinical experiences on career preference of male and female medical students. Med Teach.

[14] Lambert EM, Holmboe ES (2005). The relationship between specialty choice and gender of U.S. medical students, 1990–2003. Acad Med.

[15] Azizzadeh A, McCollum CH, Miller CC, Holliday KM, Shilstone HC, Lucci A (2003). Factors influencing career choice among medical students interested in surgery. Curr Surg.

[16] Rahman R, Zhang B, Humbyrd CJ, LaPorte D (2021). How do medical students perceive diversity in orthopaedic surgery, and how do their perceptions change after an orthopaedic clinical rotation?. Clin Orthop Relat Res.

[17] Rajani R, Haghshenas V, Abalihi N, Tavakoli EM, Zelle BA (2019). Geographic differences in sex and racial distributions among orthopaedic surgery residencies: programs in the south less likely to train women and minorities. J Am Acad Orthop Surg Glob Res Rev.

[18] Van Heest AE, Fishman F, Agel J (2016). A 5-Year update on the uneven distribution of women in orthopaedic surgery residency training programs in the United States. J Bone Joint Surg Am.

[19] Okike K, Phillips DP, Swart E, O'Connor MI (2019). Orthopaedic faculty and resident sex diversity are associated with the orthopaedic residency application rate of female medical students. J Bone Joint Surg Am.

[20] Bratescu RA, Gardner SS, Jones JM, Siff TE, Lambert BS, Harris JD, Liberman SR (2020). Which subspecialties do female orthopaedic surgeons choose and why?: Identifying the role of mentorship and additional factors in subspecialty choice. J Am Acad Orthop Surg Glob Res Rev..

[21] Hariri S, York SC, O'Connor MI, Parsley BS, McCarthy JC (2011). Career plans of current orthopaedic residents with a focus on sex-based and generational differences. J Bone Joint Surg Am.

[22] Brook EM, Hu CH, Li X, Smith EL, Matzkin EG (2020). The influence of mentors in orthopedic surgery. Orthopedics.

[23] Jurenovich KM, Cannada LK (2020). Women in orthopedics and their fellowship choice: What influenced their specialty choice?. Iowa Orthop J.

[24] Halim UA, Elbayouk A, Ali AM, Cullen CM, Javed S (2020). The prevalence and impact of gender bias and sexual discrimination in orthopaedics, and mitigating strategies. Bone Joint J..

[25] Hinze SW (2004). ‘Am I being over-sensitive?’ Women's experience of sexual harassment during medical training. Health (London).

[26] Liang R, Dornan T, Nestel D (2019). Why do women leave surgical training? A qualitative and feminist study. Lancet.

[27] Amoli MA, Flynn JM, Edmonds EW, Glotzbecker MP, Kelly DM, Sawyer JR (2016). Gender differences in pediatric orthopaedics: What are the implications for the future workforce?. Clin Orthop Relat Res.

[28] Madhuri V, Khan N (2020). Orthopaedic women of India: impediments to their growth. Indian J Orthop.

[29] Klein G, Hussain N, Sprague S, Mehlman CT, Dogbey G, Bhandari M (2013). Characteristics of highly successful orthopedic surgeons: a survey of orthopedic chairs and editors. Can J Surg.

[30] Uzoigwe CE, Middleton RG (2012). Occupational radiation exposure and pregnancy in orthopaedics. J Bone Joint Surg Br.

[31] Dineen HA, Patterson JMM, Eskildsen SM, Gan ZS, Li Q, Patterson BC, Draeger RW (2019). Gender preferences of patients when selecting orthopaedic providers. Iowa Orthop J.

[32] Mittwede PN (2020). CORR Insights®: Residency selection preferences and orthopaedic career perceptions: a notable mismatch. Clin Orthop Relat Res.

[33] Errani C, Tsukamoto S, Kido A, Yoneda A, Bondi A, Zora F, Soucacos F, Mavrogenis AF (2021). Women and men in orthopaedics. SICOT J.

[34] Gianakos AL, Mulcahey MK, Weiss JM, Samora JB, Shipley NY, Cannada LK, LaPorte DM (2022). #SpeakUpOrtho: narratives of women in orthopaedic surgery—invited manuscript. J Am Acad Orthop Surg.

[35] Zier K, Friedman E, Smith L (2006). Supportive programs increase medical students’ research interest and productivity. J Investig Med.

[36] Dusch MN, O’Sullivan PS, Ascher NL (2014). Patient perceptions of female surgeons: How surgeon demeanor and type of surgery affect patient preference. J Surg Res.

[37] Fink M, Klein K, Sayers K (2020). Objective data reveals gender preferences for patients’ primary care physician. J Prim Care Commun Health.

[38] Fram B, Bishop ME, Beredjiklian P, Seigerman D (2021). Female sex is associated with increased reported injury rates and difficulties with use of orthopedic surgical instruments. Cureus.

