# Supplementary material for: Representation of women in orthopaedic surgery: perception of barriers among undergraduate medical students in Saudi Arabia
Source: J Orthop Surg Res. 2023 Jan 7;18:19. doi: 10.1186/s13018-022-03487-6 (PMC9825039; doi:10.1186/s13018-022-03487-6)
Supplement: Supplementary file 1 — Additional file 1. Questionnaire to investigate undergraduate medical students’ perception of women participating in orthopaedic careers. [file 13018_2022_3487_MOESM1_ESM.docx]

**Supplementary file 1.** Questionnaire to investigate undergraduate medical students’ perception of women participating in orthopaedic careers

| **Questions** | **Responses** |
| --- | --- |
| **Section I: Sociodemographic Data** | |
| 1. Specify your gender: | Male/Female |
| 1. What is your marital status? | - Single - Married - Married, and I have children. |
| 1. What is your academic level? | - First year - Second year - Third year - Fourth year - Fifth year - Medical Intern |
| 1. Have you attended any orthopaedic surgery courses? | Yes/no |
| **Section II: Perception of Women in Orthopaedic Surgery** | |
| 1. Are you planning to pursue your postgraduate residency training in Orthopaedic Surgery? | Closed ended (yes/no/not sure) |
| 1. I believe that gender diversity in the field of orthopaedic surgery is an important factor contributing to job efficiency and the community of orthopaedics? | Likert ratings (1-5) |
| 1. I believe that orthopaedic surgery does not fit and is not appropriate for females. | Likert ratings (1-5) |
| 1. You believe that only particular subspecialties in orthopaedic surgery are fit for women. If you disagree, please select ‘disagree’. You can select more than one from other options:  - Disagree - I do not know - Arthroscopy and Sports Medicine - Trauma - Hand and Upper Extremity - Shoulder - Arthroplasty - Foot and Ankle Surgery - Spine Surgery - Orthopaedic oncology - Paediatric Orthopaedics - Deformity | Closed with multiple responses |
| 1. Which of the following do you believe are important barriers for women practicing in orthopaedic surgery? You can select more than one from:  - I do not believe there are barriers. - The family and social commitments. - Physical strength and body build - Gender discrimination. - The long working hours and heavy workload. - Stress and burnout. - On-call duties and covering trauma and emergency. - The radiation exposure in orthopaedics. - The patients' preference of male orthopaedic surgeon) | Closed with multiple responses |
| 1. I believe that ‘family and social commitments’ create a barrier for women practicing in orthopaedic surgery. | Likert ratings (1-5) |
| 1. I believe that the ‘physical strength and body build’ is important for practicing orthopaedic surgery and it can create a barrier for women practicing in orthopaedics. | Likert ratings (1-5) |
| 1. I believe that in orthopaedic surgery the gender discrimination exists against women and can be a barrier for them during the practice. | Likert ratings (1-5) |
| 1. I believe that the ‘long working hours and the heavy workload’ can create a barrier for women practicing in orthopaedic surgery. | Likert ratings (1-5) |
| 1. I believe that the ‘stress and burnout’ in orthopaedics can create barriers for women practicing in orthopaedic surgery. | Likert ratings (1-5) |
| 1. I believe that the on-call duties and covering trauma and emergency in orthopaedics can create a barrier for women practicing in orthopaedic surgery. | Likert ratings (1-5) |
| 1. I believe that the radiation exposure risk in orthopaedics can create a barrier for women practicing in orthopaedic surgery. | Likert ratings (1-5) |
| 1. I believe that the orthopaedic surgical abilities of male surgeons are superior to the abilities of female orthopaedic surgeons. | Likert ratings (1-5) |
| 1. I believe that patients have more confidence in male orthopaedic surgeons than females. | Likert ratings (1-5) |
